# Antimicrobial therapy in complicated rhinosinusitis

**DOI:** 10.3389/fmicb.2022.960319

**Published:** 2022-08-22

**Authors:** Simion-Antonie Catrinel Beatrice, Bejenaru Paula Luiza, Popescu Bogdan, Berteșteanu Gloria Simona, Condeescu-Cojocărița Mihnea, Cîrstea Anca Ionela, Diaconu Teodora Elena, Taher Bianca Petra, Rujan Simona-Andreea, Oașă Irina Doinița, Berteșteanu Șerban Vifor Gabriel, Grigore Raluca

**Affiliations:** ^1^Department of ENT, Head and Neck Surgery, Colţea Clinical Hospital, Bucharest, Romania; ^2^Department of Ophthalmology & ENT, “Carol Davila” University of Medicine and Pharmacy, Bucharest, Romania; ^3^Department of ENT, Head, and Neck Surgery, “Carol Davila” Emergency Central Military Hospital, Bucharest, Romania

**Keywords:** antibiotics, rhinosinusitis, complications, orbital, antimicrobial, surgery

## Abstract

**Introduction:**

Complicated rhinosinusitis is a rare but life-threatening pathology that requires both medical and surgical treatment as soon as possible. The spread of the infection from the paranasal sinuses affects, most often, the orbit, patients presenting diplopia, preseptal cellulitis, orbital cellulitis, or even blindness alongside exteriorization of puss from the middle meatus and nasal obstruction.

**Materials and methods:**

We carried out a retrospective review of 32 patients that were diagnosed in our clinic with complicated rhinosinusitis from 2015 to 2022. All the patients received at least one intravenous antibiotic, and some also received antifungal drugs. All patients underwent surgery, either endoscopically or combined approach. Nasal washout or nasal swabs during surgery were sent for laboratory examination in all patients, and we studied the microbial etiology in these extensive infections. A database with all the information regarding demographic and medical data was established.

**Results:**

78% of the patients were male, with a mean age of 50.55. A wide range of antibiotics were used, while some patients, diagnosed with mucormycosis also received antifungal drugs. The mean hospitalization period was 12 days. We correlated the type of antibiotic with the hospitalization period and the outcome but also the degree of involvement of the orbit and the microbial strains identified.

**Discussion:**

The management of patients with complicated rhinosinusitis is complex and dynamic and it must be tailored to every patient, after an interdisciplinary meeting with the infectious disease specialist, ophthalmologist, and rhinologist. The microbial strains that produce such important infections are sometimes multi-resistant or combined, patients usually already had followed a course of antibiotics at home, and choosing the right treatment is sometimes challenging.

## Introduction

Acute rhinosinusitis is one of the most frequent pathologies for which patients receive antibiotic prescriptions worldwide. Bacterial infections can lead to intraorbital or intracranial complications by direct spread, due to the proximity of the ethmoid sinus and frontal sinus to the orbit and anterior cranial fossa and the often dehiscent lamina papyracea or an anatomical abnormality at this level (Sindwani, 2021).

Acute bacterial rhinosinusitis can lead to preseptal cellulitis, subperiosteal abscess, orbital abscess, cavernous sinus thrombosis, meningitis, epidural abscess, subdural abscess, intracerebral abscess or frontal bone osteomyelitis (Sindwani, 2021).

Complicated Rhinosinusitis is a rare but life-threatening pathology that requires both medical and surgical treatment as soon as possible. The spread of the infection from the paranasal sinuses affects, most often, the orbit, patients presenting diplopia, preseptal cellulitis, orbital cellulitis or even blindness alongside exteriorization of puss from the middle meatus and nasal obstruction (Anniko et al., 2010).

Signs and symptoms of complications include eyelid edema, proptosis, impaired extraocular muscles movement, chemosis and ophthalmoplegia, headache, fever, focal neurological deficits, lethargy, or seizures. The appearance of any of these symptoms in a patient with acute rhinosinusitis should raise concern of an important complications. A detailed CT scan with contrast is mandatory and the examination must be completed by an MRI if intracranial complications are being suspected (Flint et al., 2020; Fokkens et al., 2020).

It is recommended that empiric antimicrobial therapy should be started as soon as the diagnosis of acute bacterial rhinosinusitis is confirmed. In adult patients, *Streptococcus pneumoniae*, *Staphylococcus aureus,* and *Moraxella catarrhalis* are the most frequent microbial strains identified in nasal swabs. In patients with complicated rhinosinusitis, multi-drug resistant microbial strains are usually involved (Chow et al., 2012).

Immediate and aggressive intravenous antibiotics should be started, in complicated rhinosinusitis, as soon as possible, and depending on the results of the imaging studies, exploration and drainage of the abscess are indicated in the first 24–48 h (Fokkens et al., 2020).

## Materials and methods

Between 2015 and 2022, in our clinic were admitted 35 patients diagnosed with complicated rhinosinusitis. A retrospective observational study was conducted.

In our study were included 32 patients with complicated rhinosinusitis that had given their written consent for data processing and photographic documentation. From our study were excluded patients that did not have the mental ability to give their written consent or patients that had two different pathologies (orbital or intracranial and rhinosinusitis), without proven connection.

Patients with orbital complications of acute bacterial rhinosinusitis were classified using the Chandler classification as follows: class I represents the inflammatory stage, patients presenting eyelid edema and erythema, with normal visual acuity and normal extraocular movement; in class II were included patients with orbital cellulitis. Class III is for patients with subperiosteal abscess, where there is a displacement of the globe downward or laterally, while we classified all orbital abscesses as class IV. In the situation of cavernous sinus thrombosis, according to Chandler’s classification, the patient is categorized as class V.

All the patients received at least one intravenous antibiotic, and patients that were diagnosed with fungal invasive rhinosinusitis also received antifungal drugs. All patients underwent surgery, either endoscopically or combined approach. Nasal washout or nasal swabs during surgery were sent for laboratory examination in all patients, and we studied the microbial strains involved in these extensive infections. A database with all the information regarding demographic and medical data was established.

We used Microsoft Excel 16.60 and IBM SPSS Statistics to process the data.

## Results

In this study, 32 patients were included. There were 25 males (78%) and 7 females (22%). The mean age was 50.55 years old. Most patients (83%) reside in the urban area, while 17% came from the rural areas. 75% of all patients were smokers.

The average duration of hospitalization was 12 days. We studied how many days prior presentation at the hospital the patient had symptoms of acute bacterial rhinosinusitis and the average was 14 days. In this period, 84% of the patients used amoxicillin and clavulanic acid 875 mg/125 mg twice daily, recommended by the GP and 16% used third-generation cephalosporins. Unfortunately, the use of antibiotics prior to ENT examination increases the risk of complications and the selection of microbial strains that are resistant to the first line of antibiotics. Patients often follow a short course of antibiotic and interrupt them without medical advice. It raises serious concern to the specialist that all the patients included in the study had followed prior hospitalization a course of antibiotics.

It is important to stress that in the last guidelines, third-generation cephalosporins are no longer recommended as empiric therapy in acute bacterial rhinosinusitis due to the high resistance among *S. pneumoniae*. In the past, second- or third-generation cephalosporins, doxycycline, trimethoprim-sulfamethoxazole, macrolides, and fluoroquinolones have been recommended as an alternative to amoxicillin or amoxicillin-clavulanate, but recently, a high identification of cross-resistant and multi-drug resistant strains of *Haemophilus influenzae* and *S. pneumoniae* have changed the recommendations regarding the antimicrobial agents (Chow et al., 2012).

Patients who had taken a course of antibiotics prior hospitalization are at risk of antibiotic resistance and a second-line treatment is recommended. Also, the nasal washout cultures may be sterile in spite of the fact that the disease progresses, and the symptoms worsen.

**Figure 1:**
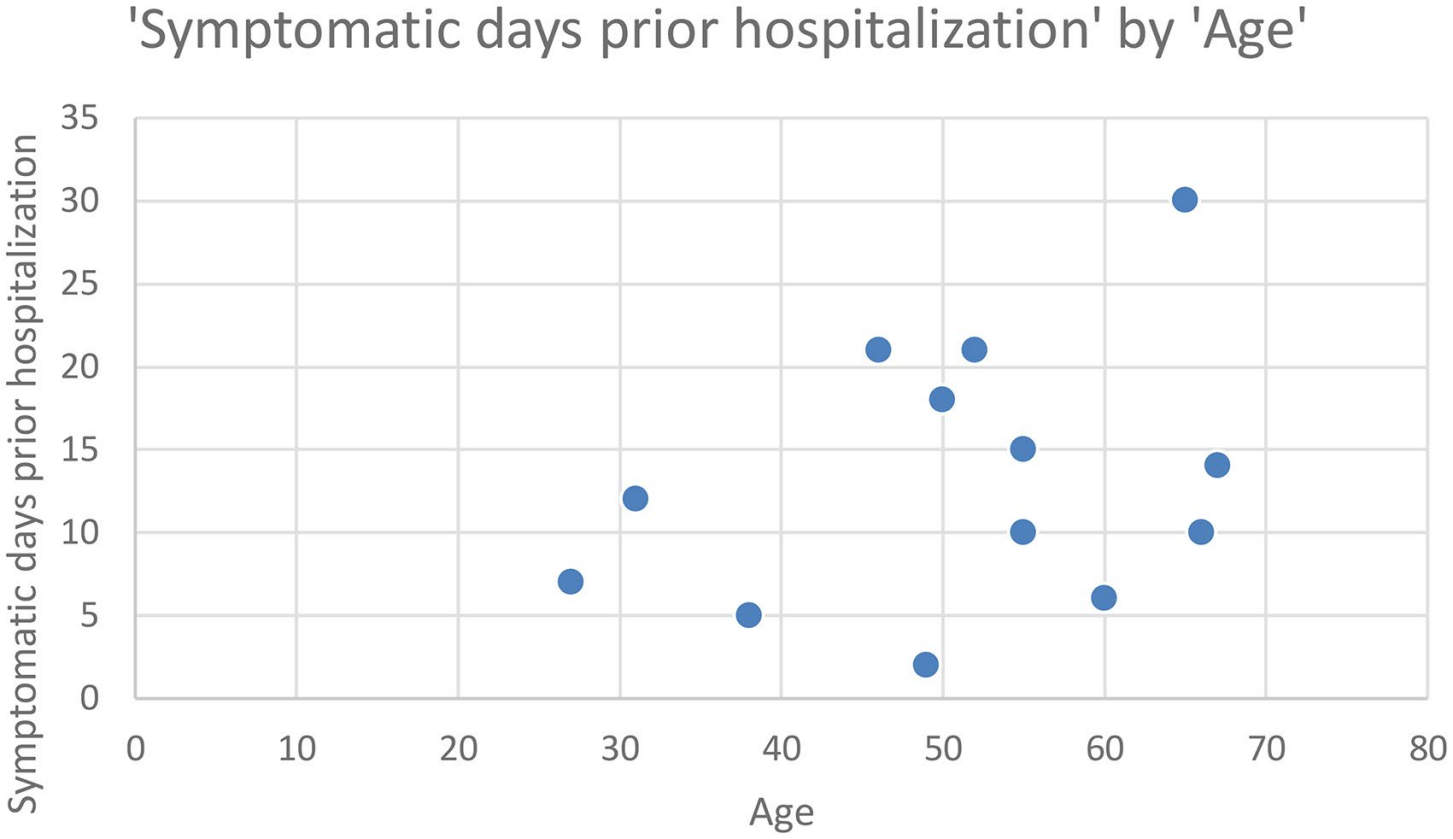
Correlation between the symptomatic days prior hospitalization and age.

Elderly patients waited longer to present to the emergency room, staying at home and self-medicating. It is important to mention, though, that 2 years of our study were the pandemic years (2020–2022), when a lot of patients avoided going to the hospital, fearing they would get infected with SARS-COV 2. In Figure 1 it is shown the correlation between the symptomatic days prior hospitalization and age – the patients that waited the longest (30 days) were between 60 and 70 years old, followed by patients between 40 and 60 years old, that waited between 15 and 20 days before presenting to the doctor.

**Figure 2:**
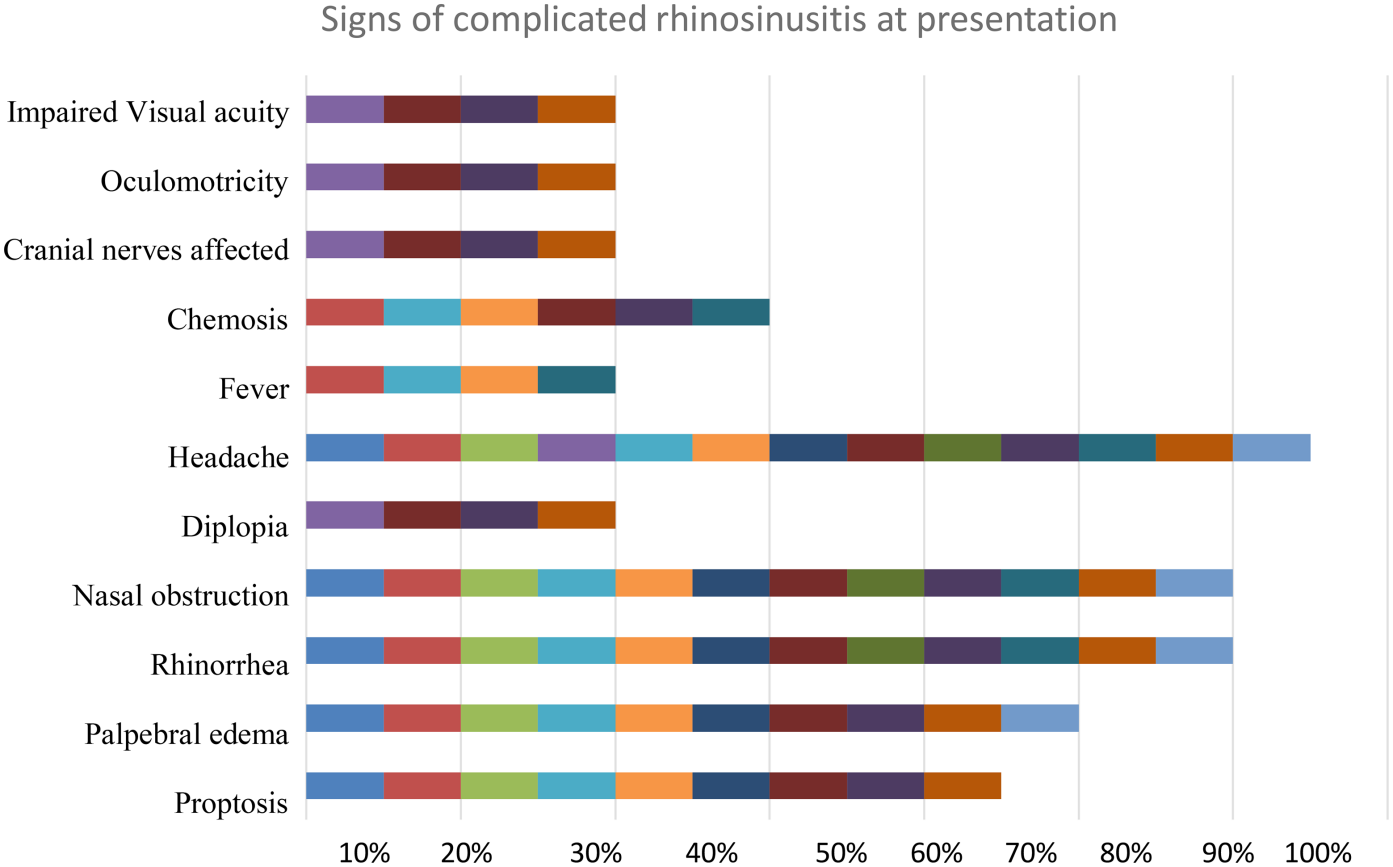
Signs of complicated rhinosinusitis at presentation.

In Figure 2 it is represented the percentage of patients that had certain signs and symptoms of complicated acute rhinosinusitis at admission to the hospital: 30% of the patients had impaired visual acuity, 30% had oculomotricity deficits, 30% had at least one cranial nerve affected by the infection, 45% of the patients presented chemosis and 30% had a history of or presented fever. 30% had diplopia at admission, this being a sign of extensive disease. Almost all patients (98%) complained of headache, this being the most common symptom. Nasal obstruction and rhinorrhea affected 90% of all patients, while palpebral edema affected 75% of all patients. Proptosis was diagnosed in 65% of all cases.

The patients that had at admission to the hospital, impaired visual acuity and oculomotricity deficits had a poor response to the medical therapy and the overall morbidity was higher than in the groups of patients without visual acuity or oculomotricity deficits.

After the clinical and endoscopical examination, all patients were assessed by computer tomography and selected cases by magnetic resonance imaging. All patients were assessed by an ophthalmologist and an infectious disease specialist and started immediate intravenous antimicrobial therapy.

Regarding the serum biomarkers, there was no statistically significant correlation between the procalcitonin levels or the C-reactive proteins at admission, nor postoperative, with the outcome or response to medical or surgical treatment.

All patients underwent surgery to drain the abscesses and open the paranasal sinuses.

Nine percent of the patients (Flint et al., 2020) had clinical and endoscopic signs of fungal disease so they also received systemic antifungal drugs. Mucormycosis is a rare but life-threatening disease, affecting the nose and paranasal sinuses, the lungs, skin, or brain. It usually affects immunocompromised patients and its incidence has risen in the pandemic era, affecting diabetic patients that have received high doses of systemic corticosteroids (Pal et al., 2021; Singh et al., 2021).

**Figure 3:**
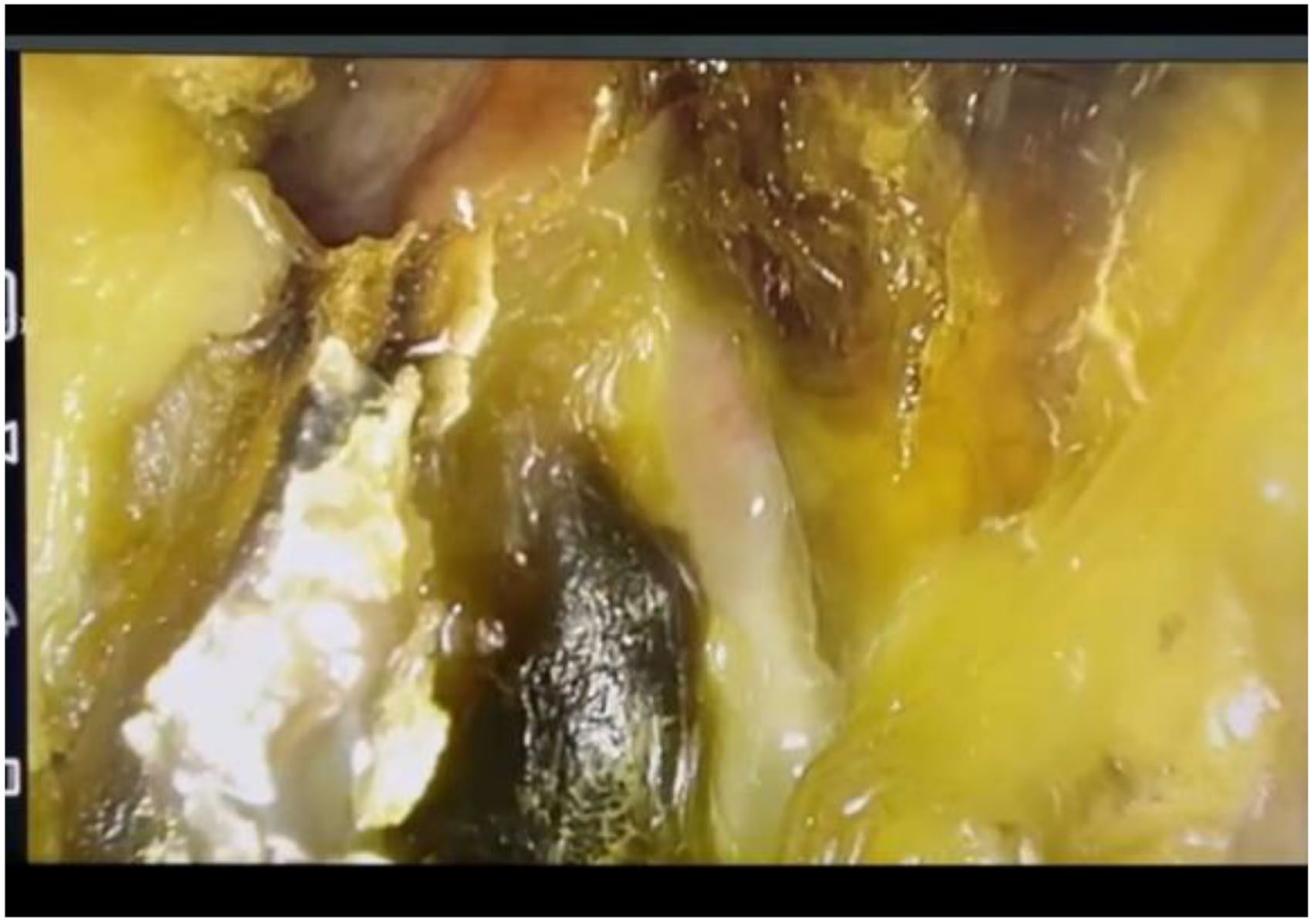
Endoscopic aspect of mucormycosis

**Figure 4:**
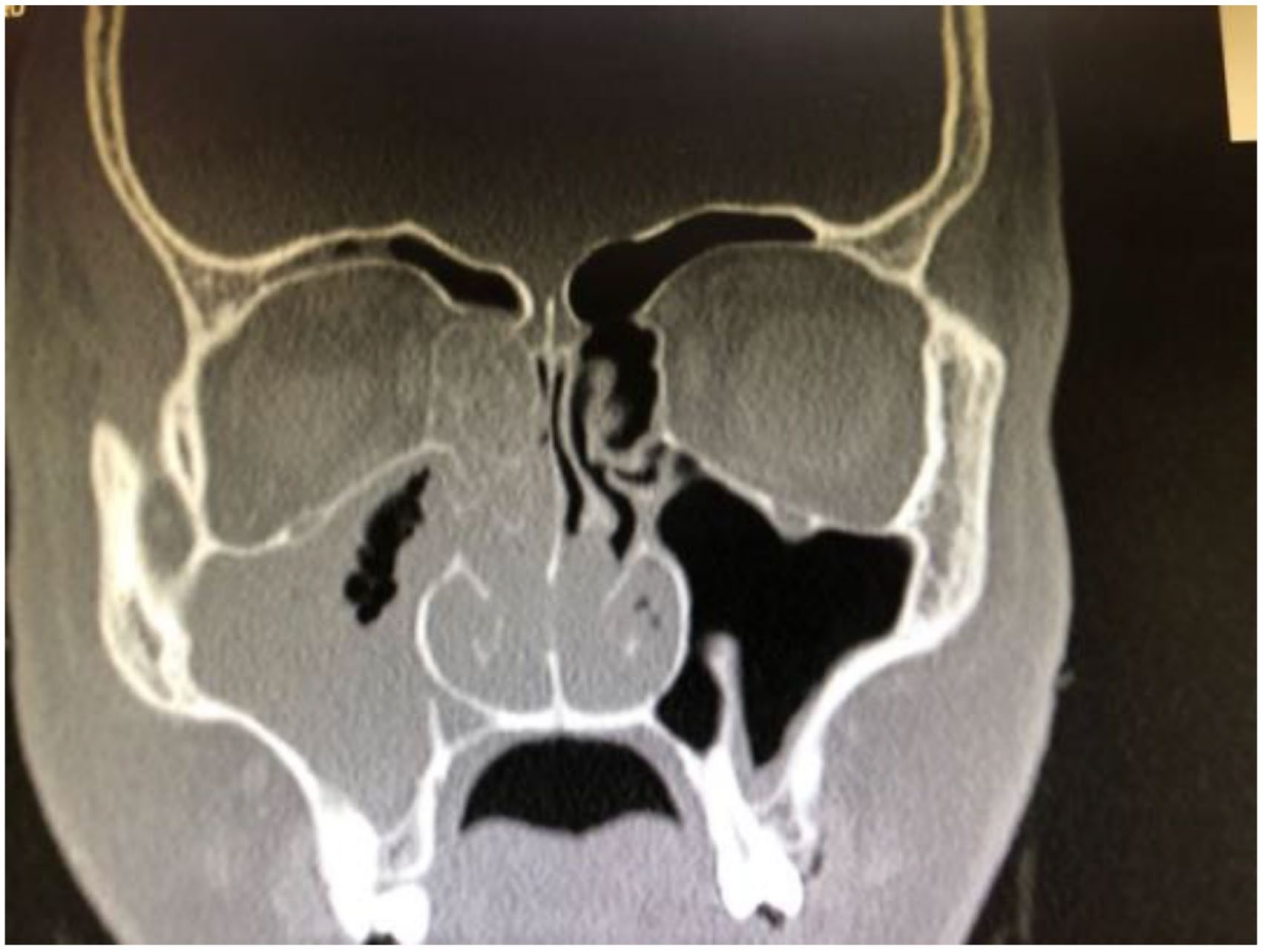
CT scan in sagittal view.

The endoscopic findings in patients diagnosed with mucormycosis are black crusts and necrosis covering the nasal mucosa as seen in Figure 3. The extent of the disease can be seen in the CT scan image selected in Figure 4.

The treatment in patients diagnosed with mucormycosis is urgent and requires urgent surgical extensive debridement and medical therapy. The treatment was initiated following the global guidelines. The first-line treatment is with high-dose liposomal amphotericin B, while intravenous isavuconazole and intravenous or delayed-release tablet Posaconazole are recommended with moderate strength (Cornely et al., 2019).

**Figure 5:**
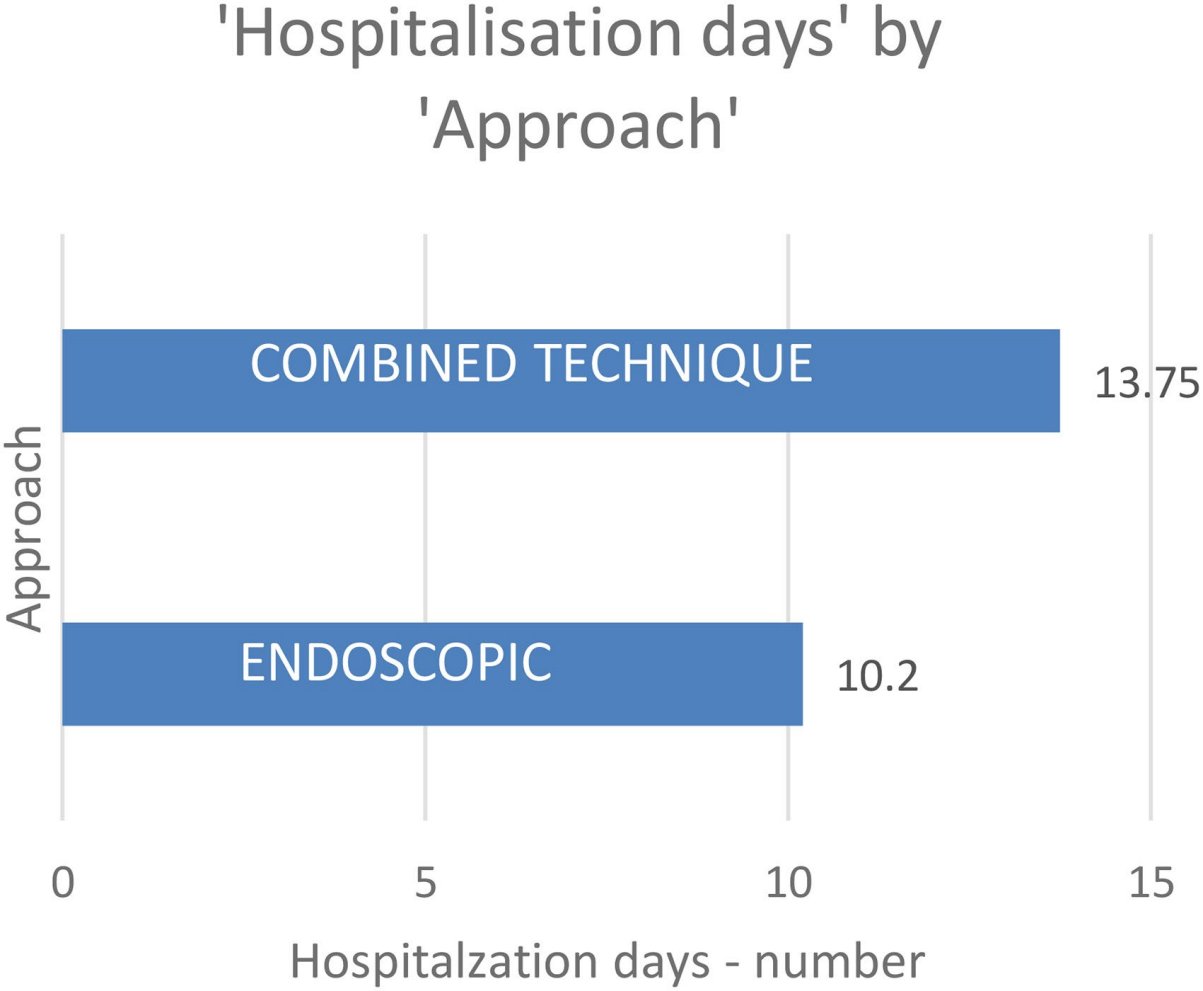
Hospitalization days by approach.

Most patients, 75% (24 patients) benefit from an endoscopic approach, while 25% of them (Cornely et al., 2019) needed a combined surgical approach, as shown in Figure 5. Patients that needed a combined technique were hospitalized for longer than those who benefit from an endoscopic treatment – patients that underwent endoscopic surgery were hospitalized for 10.2 days while patients that needed a combined technique were hospitalized for 13.75 days. The aim of the surgical treatment is to remove the thick muco-purulent sinus secretion, to obtain a culture from that secretion, and to reestablish the mucociliary flow. Also, in patients with acute complicated bacterial rhinosinusitis, the surgical drainage is mandatory in order to avoid intracerebral dissemination or other septic complications.

**Figure 6:**
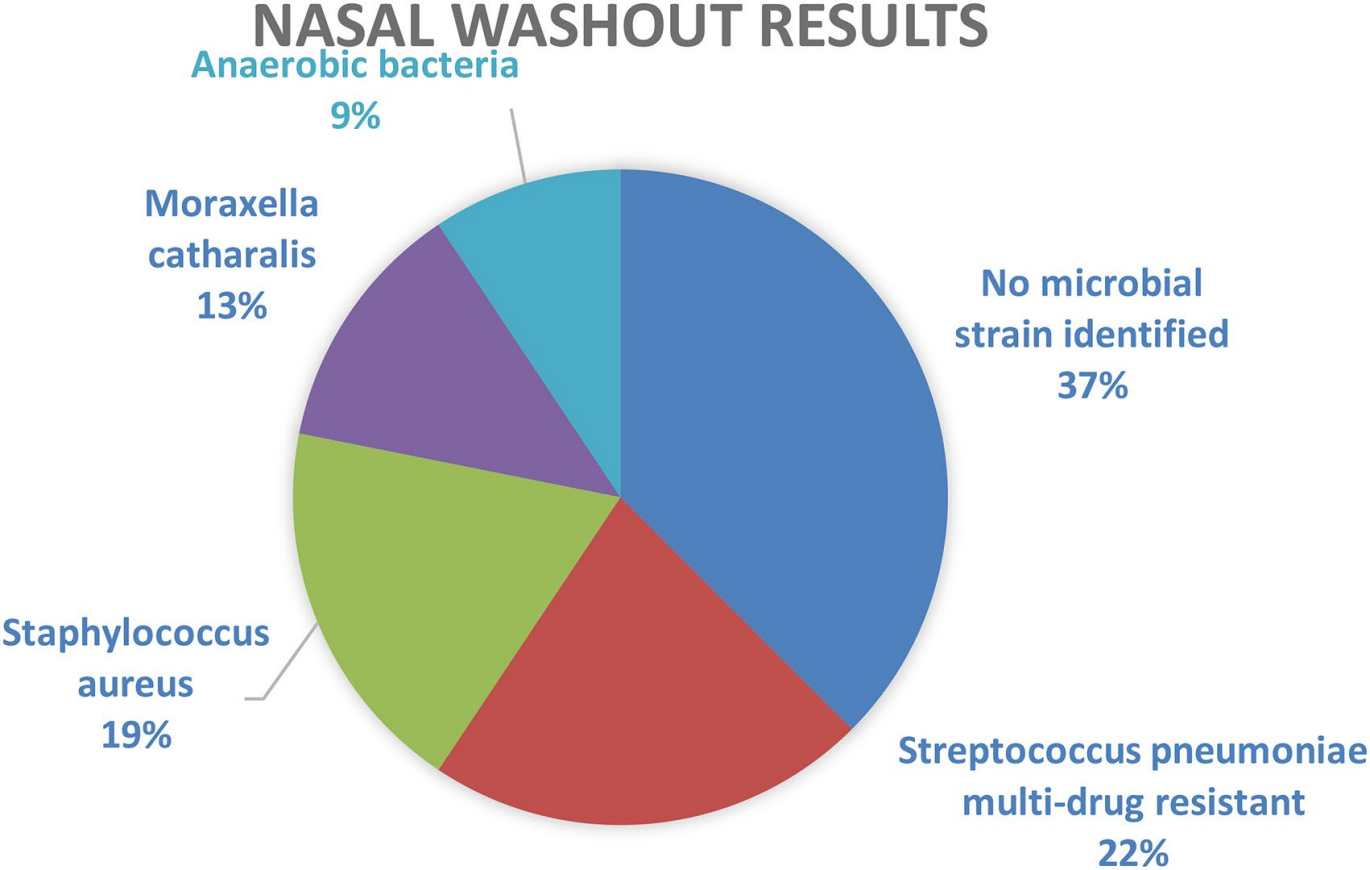
Distribution of bacterial strains isolated from the nasal washouts.

The bacteriological examination of the nasal washout was influenced by the empiric antibiotic therapy the patients had taken prior hospitalization. In 37% of cases, no microbial strain could be identified. Prior antibiotic use is a major risk factor associated with the development of infection with antimicrobial-resistant strains.

*Streptococcus pneumoniae* multi-drug resistant was identified in 22% of patients, followed by *S. aureus* in 19% of cases and *M. catarrhalis* in 13%. Anaerobic bacteria grew in 9% of the cultures.

According to Infectious Disease Society of America Guidelines, if a patient with acute rhinosinusitis has signs and symptoms that are persistent or not improving in over 10 days, are severe or worsening after 3—4 days, it is recommended the initiation of antibiotic therapy. If the patient is at risk for resistance (age under 2 or over 65, has used prior antibiotics during the last month, has had a prior hospitalization in the past 5 days, has comorbidities, or is immunocompromised), the second-line antimicrobial therapy is recommended. The respiratory fluoroquinolones have remained highly active against all common respiratory pathogens and represent the second-line antimicrobial therapy in acute bacterial rhinosinusitis. It is important to keep in mind the variety of side effects of fluoroquinolones such as seizures, headache, peripheral polyneuropathy, photosensitivity, prolongation of QT interval, and Achilles tendon rupture, among others (Chow et al., 2012).

The antimicrobial therapy was initiated for all patients at admission, in a multidisciplinary team formed by the ENT surgeon, infectious disease specialist, and intensive care doctor. All patients received intravenous antimicrobial therapy, 85% receiving a combination of two antibiotic classes, usually one directed to anaerobic microbial strains. The patients that had a high suspicion for invasive fungal disease received antifungal intravenous treatment.

The international guidelines recommend for patients with acute complicated bacterial rhinosinusitis, the initiation of the second line of antimicrobial therapy, either with intravenous fluoroquinolones or with a combination of third-generation cephalosporin and clindamycin or doxycycline, bearing in mind the high risk of *Clostridium difficile*-associated enterocolitis (Chow et al., 2012).

Most patients received intravenous Levofloxacin (56%) while 23% received a regime of combination therapy of Clindamycin plus Cefixime. 21% of patients received intravenous Linezolid. Patients that had a result of anaerobic bacteria at the nasal washout received Metronidazole.

All patients survived and were discharged from the hospital. Three patients (9%) lost their vision in one eye completely due to extensive disease at the moment of admission in the hospital.

## Discussions and conclusions

The management of patients with complicated rhinosinusitis is complex and dynamic and it must be tailored to every patient, after an interdisciplinary meeting with the infectious disease specialist, ophthalmologist, and rhinologist. The microbial strains that produce such important infections are sometimes multi-resistant or combined, patients usually already had followed a course of antibiotics at home, and choosing the right treatment is sometimes challenging. If an intracranial complication is suspected, double antibiotic coverage with a third-generation cephalosporin and metronidazole at an appropriate dosage should be initiated.

Regarding the antimicrobial therapy, the international guidelines recommend taking into consideration prior antibiotic exposure, the severity of the disease, and the rate of the progression of the disease (Anon et al., 2004).

Fluoroquinolones represent the second line of treatment in acute bacterial rhinosinusitis and is the antibiotic of choice in complicated cases, but it is important to keep in mind that limiting the overuse of this particular class of antimicrobial drugs may help in reducing the bacterial resistance (Chow et al., 2012).

Macrolides and trimethoprim/sulfamethoxazole are not recommended in the guidelines in the treatment of acute bacterial rhinosinusitis due to the high percentage of resistant strains of *S. pneumoniae* and *H. influenzae*.

When choosing the antimicrobial therapy for a patient diagnosed with acute complicated rhinosinusitis, who is at risk for antibiotic resistance, it is important to evaluate all the risks and benefits of that particular drug and to discuss all the options presented in international guidelines, in a multidisciplinary team, so that the best therapy for each particular patient is selected.

Serum inflammatory biomarkers, such as procalcitonin and C-reactive protein are promising, but their levels do not always correlate with the patients’ outcome. These biomarkers have been studied as an indication for antibiotic therapy initiation, but the conclusions are not firm.

There are discussions regarding the timing of the surgical drainage. “Immediate” drainage is not well defined, but usually, there is accepted a window of 24–48 h in which the patient should be properly assessed.

In our study were included only patients with extensive, complicated rhinosinusitis, that required aggressive antimicrobial treatment. Also, all of them had followed a short course of oral antibiotics, either amoxicillin with clavulanic acid, or cephalosporins. This has been an important factor in choosing the antimicrobial therapy in hospital.

The SARS-COV 2 infection has risen the incidence of complicated acute bacterial rhinosinusitis, partly because patients presented at the hospital late. Also, the incidence of invasive opportunistic fungal disease has increased during the pandemic, especially in diabetic patients that received high doses of systemic corticosteroids.

## Data availability statement

The original contributions presented in the study are included in the article/supplementary material, further inquiries can be directed to the corresponding author.

## Ethics statement

Written informed consent was obtained from the individual(s) for the publication of any potentially identifiable images or data included in this article.

## Author contributions

All authors listed have made a substantial, direct, and intellectual contribution to the work and approved it for publication.

## Conflict of interest

The authors declare that the research was conducted in the absence of any commercial or financial relationships that could be construed as a potential conflict of interest.

## Publisher’s note

All claims expressed in this article are solely those of the authors and do not necessarily represent those of their affiliated organizations, or those of the publisher, the editors and the reviewers. Any product that may be evaluated in this article, or claim that may be made by its manufacturer, is not guaranteed or endorsed by the publisher.
